# The use and limits of scientific names in biological informatics

**DOI:** 10.3897/zookeys.550.9546

**Published:** 2016-01-07

**Authors:** David Remsen

**Affiliations:** 1 Department of Marine Resources, Marine Biological Laboratory, 7 MBL Street, Woods Hole, MA 02543

**Keywords:** Taxonomic name services, taxon concepts, identifiers, relevance, search and retrieval

## Abstract

Scientific names serve to label biodiversity information: information related to species. Names, and their underlying taxonomic definitions, however, are unstable and ambiguous. This negatively impacts the utility of names as identifiers and as effective indexing tools in biological informatics where names are commonly utilized for searching, retrieving and integrating information about species. Semiotics provides a general model for describing the relationship between taxon names and taxon concepts. It distinguishes syntactics, which governs relationships among names, from semantics, which represents the relations between those labels and the taxa to which they refer. In the semiotic context, changes in semantics (i.e., taxonomic circumscription) do not consistently result in a corresponding and reflective change in syntax. Further, when syntactic changes do occur, they may be in response to semantic changes or in response to syntactic rules. This lack of consistency in the cardinal relationship between names and taxa places limits on how scientific names may be used in biological informatics in initially anchoring, and in the subsequent retrieval and integration, of relevant biodiversity information. Precision and recall are two measures of relevance. In biological taxonomy, recall is negatively impacted by changes or ambiguity in syntax while precision is negatively impacted when there are changes or ambiguity in semantics. Because changes in syntax are not correlated with changes in semantics, scientific names may be used, singly or conflated into synonymous sets, to improve recall in pattern recognition or search and retrieval. Names cannot be used, however, to improve precision. This is because changes in syntax do not uniquely identify changes in circumscription.

These observations place limits on the utility of scientific names within biological informatics applications that rely on names as identifiers for taxa. Taxonomic systems and services used to organize and integrate information about taxa must accommodate the inherent semantic ambiguity of scientific names. The capture and articulation of circumscription differences (i.e., multiple taxon concepts) within such systems must be accompanied with distinct concept identifiers that can be employed in association with, or in replacement of, traditional scientific names.

## Introduction

Scientific names are labels for taxa that are governed by formalized rules of nomenclature. These rules were introduced to establish clarity, stability, economy and uniqueness to the fragmented landscape of pre-Linnaean nomenclature (Mayr 1991). Sherborn’s *Index Animalium*
(IA) represents a monumental attempt to capture key data elements regarding the source and orthography of (nearly) all zoological names for species from the beginning of formalized Linnaean zoological nomenclature in 1758 through 1850. An index, in the sense of *Index Animalium*, is a list of terms linked to, or pointing to, a greater volume of values, data, information or knowledge that pertain to the term. *Index Animalium* links zoological names to their originating bibliographic citation. (Pilsk et al. 2016) It also links two separate records when a species described with one name was subsequently moved to a new genus and established a new binomial name. The primary function, of the more than 9,000 pages, however, is as an authoritative reference that provides the correct spelling of a name and pointer to its original description (CWR 1903, Alonso-Zarazaga et al. 2016). Much of the value and respect that IA has received is derived from the enormous amount of work required to compile and verify the names and associated publications. Biologists rely on this reference when they need to consult the original work (Evenhuis 2016).

The use and value of IA, however, extends beyond its referential value and, in an age of increasingly vast amounts of digitized biodiversity information being accessible online, serves as an immensely valuable resource in establishing order within a substantially larger index of biodiversity information: the entire corpus of recorded biodiversity knowledge.

Throughout the past 250 years, nearly all information about taxonomic groups such as species has been linked through a name, nearly always a scientific name. (Grimaldi and Engel 2005). Scientific names are not the sole means to label species information. Informal and provisional names also play supporting roles (Murray and Stackebrandt 1995) but only when their use can be unambiguously linked to a species through a scientific name. Practically speaking, scientific names form the basis for referring to species and they label biodiversity information across the entire spectrum of biodiversity knowledge (Thompson and Pape 2016).

**Figure 1.**
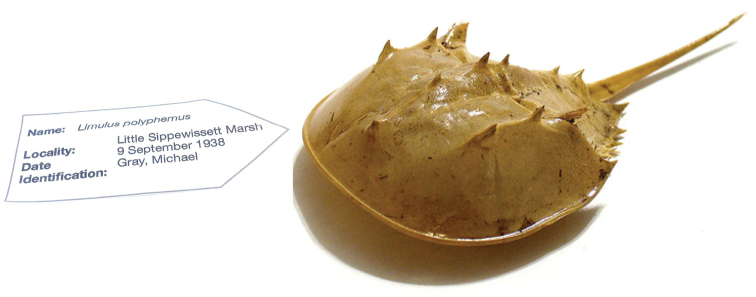
Scientific names label information about species.

Names label voucher specimens in natural history museums, for instance, and are used to identify biological observations at all scales, from molecules to ecosystems, providing the key biological context to associated metacontent such as the observation locality and date Figure 1. Scientific names are used in all manner of publications and communications be they scientific, agricultural, commercial, medical, legislative or social. Increasingly, these communications are taking place online and in digital environments and legacy information is being retrospectively digitized and also placed in online data stores (Patterson et al. 2010). Without a name associated with an information or data object, the taxonomic link is effectively lost.

## Discussion

Given the ubiquitous linkage between biodiversity information and scientific names, there must exist an enormous and virtual super-index of names tied to the world’s species information. Such an index, assembled and presented within a Sherborn-like data store, would, in principle, link to all, or nearly all, information related to all described species. This implies a far more important and central role for names as mediators to biodiversity information. As more and more retrospective and prospective information is placed in online data stores, such an index is becoming increasingly realistic (Pyle 2016).

Indexing and search engines like Google and Yahoo generate billions of dollars in revenue by processing countless electronic data stores and producing searchable indexes (Varian 2007). An index of names, such as provided through *Index Animalium*, combined with similar technologies could, in principal, provide access to whatever online information is associated with the names in the index. Broaden the list through the consolidation of similar indexes within the zoological, microbial and botanical domains, and it’s not inconceivable that a comprehensive list of names could be assembled. Such a collection would provide the means to discover and access the complete wealth of recorded biodiversity knowledge (Patterson et al. 2010). The legacy of Linneaus and Sherborn appear to have provided the framework for the systematic organization and delivery of biodiversity knowledge in the digital age (Patterson et al. 2006).

There are limits to this utility however, and these limits are inherent within biological nomenclature and its relationship to the taxa they label. *Index Animalium* and other similar compilations represent a list of names, not lists of species. This distinction has ramifications that place limits on helping us utilize their ubiquity in labeling biodiversity information. Semiotics refers to the study of how we use signs or symbols, such as names, to confer meaning to objects in the real world and provides a broader framework to this understanding. Semiotics is divided into several sub-domains that include:

- *Semantics*, which refers to the relationship between signs and the things to which they refer; their meaning.
- *Syntactics*, which refers to the relationships among signs or symbols within formal structures.

These two terms have analogs in taxonomy. Nomenclature, particularly formalized scientific nomenclature governs much of the syntax domain while semantics is the realm of taxonomy, which links names with taxon definitions or *circumscriptions* (Witteveen 2014, Dubois 2005, Franz et al. 2008).

The triangle of reference, or semiotic triangle (Ogden and Richards 1923), is a model of how syntax and semantics are related to the objects they represent (van Rijsbergen 1979).

In the model (Figure 3), there is no direct relationship (dotted line) between symbols (i.e., *names*) and the real-world objects (the *referent*) they represent (A). Meaning, or the relationship between the name and the object, is conveyed only through a concept that exists in the mind of the user of the name. In taxonomy, a biologist (B) determines a specimen is sufficiently distinct to constitute a new species and documents the concept or idea of this novelty to a publication and assigns a name to it. Another person (C) subsequently reading the name, perhaps as a label on a specimen, evokes the concept originally described by the biologist, to refer to the specimen. Accurate communication occurs when there is congruence between both concepts among the writer and the reader.

**Figure 2.**
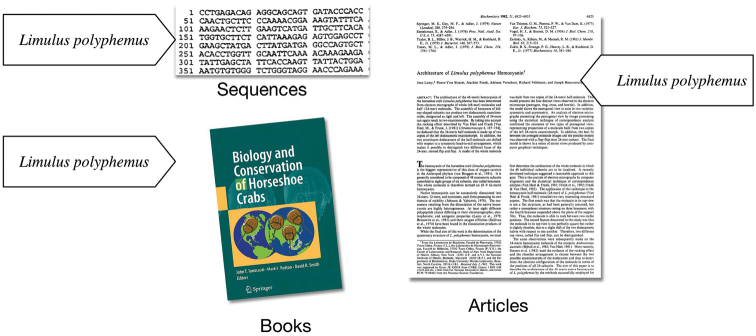
All information of a species is linked by a name.

**Figure 3.**
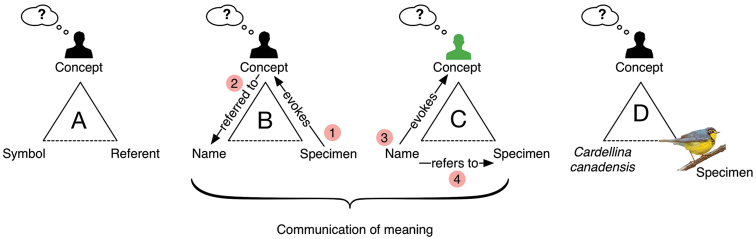
The semiotic triangle describes how names communicate meaning.

In biological taxonomy, a species name refers to a concept anchored by a specimen but created in the mind of a biologist. The function of the name is to facilitate communication. Communication is facilitated, however, only when the concepts (not the objects) are approximately congruent. Success is not black and white, but can be partial – whether partial is good enough is contingent on context-specific inference needs that the reciprocal concept alignment must fulfill. Thus, two persons look at the same avocado and one declares it a fruit, because it is derived from floral ovaries, while another declares it is not a fruit because it is not sweet. This conflict occurs when there is no congruency in the concepts invoked through the use of the name. Similar issues occur within taxonomy. In the simple case above, the term ‘fruit’ is associated with two definitions, or, more formally, the *cardinality* between syntax and semantics is one-to-two, or more generally, one-to-many (1:N). The same object evokes the same name but refers to two concepts according to two individuals. It is the relationship between the name and the concept that is important. Cardinality between syntax and semantics has a direct impact on the use and limits of scientific names as identifiers in biological informatics (Franz NM 2014).

Identifiers such as names have utility in information discovery and retrieval that is directly proportional to the degree of correlation between the term and the associated meaning or, in the semiotic context, in the correlation between syntax and semantics. Laypersons may think of scientific names as stable and unique, where a single Latin binomial name refers to one species and remains that way for all time. In other words, that there is a stable one-to-one relationship between a name (syntax) and the taxon (semantics) that it labels (Stringer 2002). This is an important informatics pre-condition if we are to rely on names as a means to search for and retrieve relevant information related to taxa (Thau et al. 2010).

**Figure 4.**
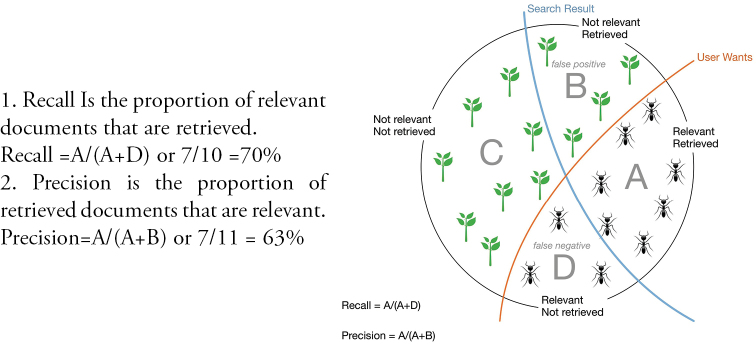
Precision vs. recall in search results.

Relevance in information retrieval is measured as a combination of two factors: precision and recall (van Rijsbergen 1979). Precision refers to the exactness, or quality in an information retrieval instance. Recall is a measure of quantity or completeness. For example, in Figure 4, below, a search for articles on ants misses some relevant articles but also accidentally returns articles on plants.

A search result, therefore, can produce two kinds of relevance errors.

- A false positive error occurs when the system returns a result that is non-relevant. This is an error of precision.
- A false negative error occurs when the system fails to return a relevant result. This is an error of recall.

### Perfect identifiers

Based on the above, we can define a perfect identifier as one that returns 100% relevant results; that is, zero false positive, and zero false negative, results. This is easy to understand in a relational database system that uses internal unique identifiers to ensure that all relevant records are returned in queries. Relational integrity within a database management system relies on a 1:1 relationship between a primary key and the object it represents. Integrity would be lost if two identifiers referred to the same object or if the same identifier referred to two objects. For an identifier to be a perfect identifier both the cardinality and correlation between syntax and semantics is exactly 1:1. From a taxonomic standpoint, this would require a single, unique name to refer to a single, distinct taxon. Any change or difference in semantics should be linked to a corresponding change in syntax (Lepage et al. 2014). This is not, however, the reality of biological taxonomy (Franz et al. 2008, Berendsohn and Geoffroy 2007).

Laypersons are often surprised to learn that scientific names are neither stable nor unique identifiers for taxa. The underlying causes for this instability have their roots in both syntax and semantics (Witteveen 2014) but the common consequence is a departure from the tight 1:1 correlation that is required to maintain the relational integrity between a name and the taxon to which it refers. This cardinal relationship dictates the utility and application of scientific names within biological information retrieval. It is this relationship between syntax and semantics that dictates whether the impact on relevance will fall on precision or on recall, or on both.

There are four cardinal relationships possible between syntax (names) and semantics (taxa) in this regard and they are summarized in Table 1 below.

**Table 1.** Summary of cardinal relationships between names and taxa.

				Impact	
Cardinality	Abbrev.	Diagram	Example	Recall	Precision
One to One	1:1	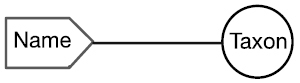	Stable taxon	No	No
Many-to-One	N:1	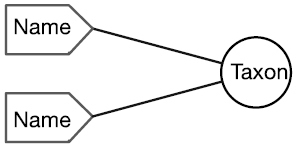	Synonyms	Yes	No
One-to-Many	1:N	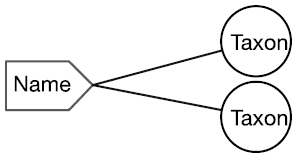	Homonyms/ Polysemes	No	Yes
Many-to-Many	N:N	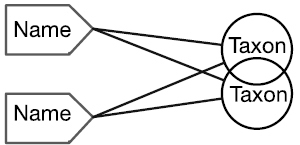	Taxon Concept	Yes	Yes

The relationship between a scientific name and the taxon to which it refers always falls into one of the four conditions in this table. Each of these conditions is represented within biological taxonomy and imposes informatics challenges that, in many cases, may be mitigated.

**One-to-One:** 

One-to-One (1:1)	Cardinality		Impact	Result
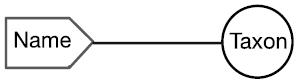	Syntax	Semantics	No impact on precision/recall	Maximum relevance
	One Name	One Meaning		

The perfect identifier, as defined above, returns no false positive or false negative results when applied in a search. Thus, a search by name returns all and only the relevant related objects. In biology, there are many taxa that are so under-studied that they are only known from their original description and none or very few subsequent references (Thessen et al. 2012). The name alone, so long as it is a unique name, is sufficient to locate all related material. As noted, under these conditions:

- Recall – A single name will ensure no false negatives will be missed
- Precision – A single taxon labeled with the name will ensure no false positives are included in the results.

In reality Latinized scientific names are complex and easily misspelled such that this pure one-to-one condition is not as easily met. When this occurs, multiple synonyms refer to the same taxon.

**Many-to-One:** 

Many-to-One (N:1)	Cardinality		Impacts	Result
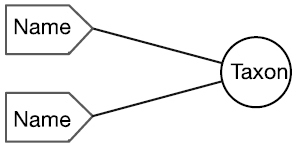	Syntax	Semantics	Recall	False negatives
	Multiple names	One meaning		

Synonyms are multiple names associated with a single taxon. Rules of nomenclature dictate that only one name is the correct label for a taxon. Any others must be “sunk” in synonymy (Del Hoyo and Pedrola-Monfort 2010). When this occurs it is clear that a single name may no longer be used to retrieve all information related to the taxon. This has the following impact on relevance.

- Recall – Synonyms impact recall because the use of a single name will result in false negative results.
- Precision – Synonyms in a N:1 condition do not impact precision because, by definition, only a single concept is involved. Thus, false positive results are not possible through matching any of the names.

**Synonyms fall into several classes based on their origin**:

Orthographic or lexical synonyms

Variations in spelling represent one class of synonyms although they are often not formally referred as such. The names “*Loligo pealeii*,” “*Loligo pealei*” and “*Loligo pealii*” for example, have all been used to refer to a particular species of squid (*Loligo pealeii* Laseur, 1821) although only the first spelling is correct. “*Pomatomus saltator*” and “*Pomatomus saltatrix*” represent variants based on differences in Latin gender applied to the species name. While only one is syntactically correct, they are both regularly used (David and Gosselin 2002). (Welter-Schultes et al. 2015) This conflation of syntax impacts recall when data stores containing variant orthographies are searched.

Nomenclatural synonyms

Nomenclatural synonyms represent a syntactic change without an associated change in semantics. This may occur when two names are discovered to refer to the same original publication or to the specimens that form the basis for the description. For example, the name *Taraxacum officianale* F.H. Wigg shares the same type specimen as the name *Leontodon taraxacum* L. The two names therefore, refer to the same taxon (Kirschner and Štěpánek 2011).

The binomial name of scientific names result in a change in syntax when a taxon is moved to a different genus or if a name is not published according to formal nomenclatural rules (Blackwelder 1967, Dubois 2000, International Commission on Zoological Nomenclature 1999, McNeill et al. 2005). When *Drosophila melanogaster* was proposed to belong within the genus *Sophophora*, a new binomial, *Sophophora melanogaster* was created. These syntactic changes are not reflective of a change in taxonomic circumscription (van der Linde and Yassin 2010).

### Taxonomic synonyms

Taxonomic synonyms are the result of a change in circumscription that occurs when two, formerly distinct taxa, are merged. This may occur due to broad variation within a species giving rise to multiple, correctly published species descriptions that are ultimately deemed to belong to the same taxon. For example, *Antilocapra anteflexa* Gray, 1855, is an antelope who’s description was based on a pair of horns. It has since been determined, and is now generally accepted to be, a variant of a previously-described species, *Antilocapra americana* Ord, 1815 (O. Gara 1978). Syntactic and pragmatic rules result in one name being applied to the newly merged taxon while other names, which may include orthographic variants and nomenclatural synonyms linked to the grouped taxa, fall into synonymy (Polaszek 2008).

In all of these cases, information tied to a single taxon may be labeled with multiple different labels. This will result in false negative results in search and retrieval across data stores containing multiple names for the taxon.

### Mitigation of synonyms

Different approaches have been applied to overcome the impact on recall inherent to synonymy.

- “Fuzzy” name-matching services are used to group orthographic variants and misspellings (Rees 2014).
- Taxonomic names servers, such as provided by uBio, iPlant and ITIS offer thesaurus-like services that provide the list of related names that can be used to conflate a search and improve recall (Remsen 2014, Boyle et. al. 2013) (Integrated Taxonomic Information System 2014).

### Homonyms

**One-to-Many:** 

One-to-Many (1:N)	Cardinality		Impacts	Result
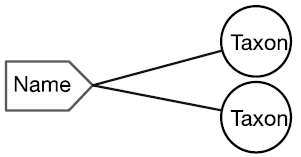	Syntax	Semantics	Precision	False positives
	One name	Multiple meanings		

Homonyms are two identically-spelled names that refer to two distinct taxa. For example, the genus *Aotus* refers to both a legume and a primate (Remsen and Patterson 2010). The species *Agathis montana* may be either a wasp or a conifer (Encyclopedia of Life 2014). Secondary homonyms are a consequence of transferring a species to a new genus that already contains the constructed binomial (Dickinson 2016).

The word *homograph* is similarly used to refer to two identically-spelled words and broadens the definition outside of biological taxonomy. *Cancer*, for example, is both a genus of crab and a medical condition. The result, however is the same: a one-to-many (1:N) relationship between syntax (one name) and semantics (two taxa). This has the following impact on relevance:

- Recall – Homonyms do not impact recall in this condition, because, by definition, only a single name is relevant and false negative matches are not possible.
- Precision – Homonyms impact precision because the name is ambiguous and can produce false positive results when a match is made to a non-target taxon.

### Mitigation of homonyms

There are two ways to improve precision when a name is too ambiguous; syntactic and pragmatic. The syntactic approach is to change the cardinality between the names and taxa from one-to-many to one-to-one. This is achieved by changing the syntax to two distinct forms. In the case of *Aotus* for example, the legume may be formally referred to as *Aotus* Smith, 1805 while the primate is *Aotus* Illiger, 1811. While the name components remain identical, the appended authorship information renders them distinct. The usage of this form of the name improves precision, effectively moving the burden of relevance to recall. The use of the more precise term supports the distinction of the two taxa but results in the minting of a synonym. The result would be fewer false positive but a potential increase in false negatives as the use of the more refined name would miss relevant results labeled only with the homonym. In general, improvements in precision result in a decrease in recall and vice-versa; Resolving this would require changing all the retrospective ambiguous use of the name *Aotus* with the more precise amended form (Rees 2012).

The pragmatic approach relies on analytic techniques that try to identify context to disambiguate the term. For example, the term “monkey” or “pea” in the vicinity of the use of the name *Aotus*, could help disambiguate the usage and improve precision (Boulis and Ostendorf 2005).

**One-to-Many:** 

One-to-Many	Cardinality		Impacts	Result
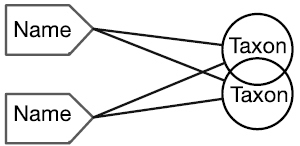	Syntax	Semantics	Precision	False positives
	One name	Multiple meanings		

Polysemy (literally “many meanings”) is a condition similar to homonymy and refers to a single name that refers to two taxa. Instead of consisting of entirely distinct taxa, however, the circumscriptions overlap. This occurs when taxa are lumped and split and result in two or more taxon concepts (Remsen and Patterson 2010) (Franz and Cardona-Duque 2013). A polyseme impacts relevance in a manner similar to a homonym but is much more common. Impacts on relevance are as follows:

- Recall – Recall is not impacted. Syntactic ambiguity is not a factor here as there is only a single name.
- Precision – Polysemes impact precision because a single taxon name refers to two or more different circumscriptions for a taxon.

**Figure 5.**
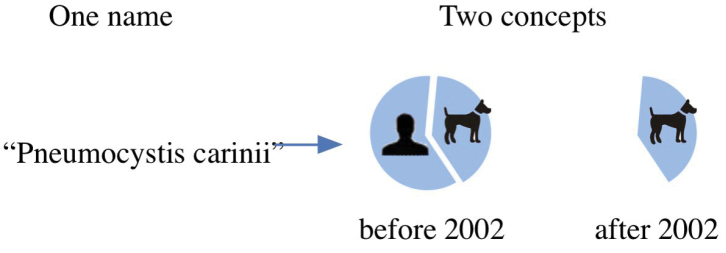
A polyseme is a single name referring to more than one overlapping or included concept.

*Pneumocystis carinii* Delanoe & Delanoe, 1912, is a fungal pathogen responsible for a deadly pneumonia (PCP) in HIV-infected patients. It was originally described in dogs and rats and later found to occur in humans. In 2002 genetic analysis determined that the human pathogen was distinct from the one that infects dogs. A new name, *Pneumocystis jiroveci*, was applied to the human pathogen (Stringer 2002). The result was a taxonomic split where the original name, *Pneumocystis carinii* subsequently referred to just part of the original circumscription and the new name *Pneumocystis jiroveci*, to the remaining part. Figure 5 illustrates this split.

The name, *Pneumocystis carinii*, now refers to two different circumscriptions; one that contains dog and human pathogens and one that contains only dog pathogens. A search using the name can return results that may refer to either use of the name, corresponding to false positives that impact precision.

### Mitigation of polysemes

Rules of nomenclature do not support reflective syntax changes due to changes in circumscription. When a taxon is split, the original name is carried on to refer to one of the resultant parts.

(Berendsohn W. G. 1995) has suggested that the name be concatenated with the annotation “sensu” followed by the author of the split to denote the circumscription reference with a unique label. In this case, the taxon would be known by two names:

- “*Pneumocystis carinii* Delanöe & Delanöe, 1912” (original) or plain “*Pneumocystis carinii*”.
- “*Pneumocystis carinii* sensu Stringer, 2002” (new).

This syntax would provide the means to distinguish the two circumscriptions in any future application but it leaves all previous applications ambiguous since the earlier application of the name can, in the context of the subsequent split, refer to either of the two new concepts. Any previous applications of the name would have to be re-assessed and re-labeled for any retrospective precision improvements. In some cases, this can be inferred through re-inspection and reasoning, using both manual and automated methods (Lepage et al. 2014). For example, pre-2002 medical literature referring to *Pneumocystis carinii* in human patients can be reasonably inferred to refer to the more precise taxon, *Pneumocystis jiroveci*.

**Many-to-Many:** 

Many-to-Many	Cardinality		Impact	Result
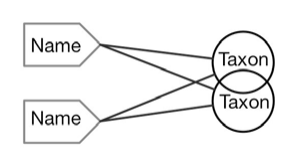	Syntax	Semantics	Precision & Recall	False positives False negatives
	Many	Many		

Polysemes were introduced as referring to a single name referring to multiple, related taxa; a condition that results from splitting a taxon concept into two or more new circumscriptions. Polysemy, however, is not the only result of semantic changes. When *Pneumocystis carinii* was divided into the human pathogen, *Pneumocystis jiroveci* and a reduced, non-human-infecting *Pneumocystis carinii*, the net result was three distinct circumscriptions labeled with two names.

**Table 2.** The result of a taxonomic split on syntax and semantics.

Syntax		
*Pneumocystis carinii*	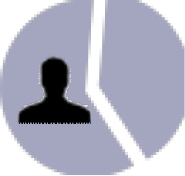	Original taxon that infects both dogs and humans
	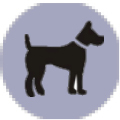	New taxon that only infects dogs
*Pneumocystis jiroveci*	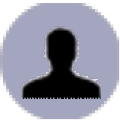	New taxon that only infects humans

The relationship between the names and the circumscriptions corresponds to a many-to-many (N:N). The two names and three concepts are all inter-related (Franz N. M. 2014).

The net result of the split, and the resultant impact on relevance in search, is summarized in Table 3 above. Synonymy can be used to positively improve precision and recall for the lumped taxon when applied to search and retrieval. When taxa are split, synonymy may improve recall by retrieving otherwise false negative results tied to the use of synonyms. As one of the names is a polyseme, however, synonymy cannot improve precision.

**Table 3.** Lumped and split taxon and use of names to impact relevance where P=Precision and R=Relevance.

Taxon infects	Names	Semantics	P	R
*Dogs and humans*	***Pneumocystis carinii*** - Synonym: *Pneumocystis jiroveci*	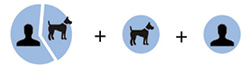	Y	Y
*Dogs only*	***Pneumocystis carinii*** - Syn.: *Pneumocystis carinii* (part)	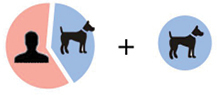	N	Y
Humans only	***Pneumocystis jiroveci*** - Syn.: *Pneumocystis carinii* (part)	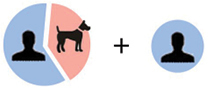	N	Y

## Summary

Scientific names link nearly all information related to a species but the relationship between nomenclatural syntax and taxonomic semantics is inherently ambiguous. Informatics processes that rely on data-gathering methods linked to taxon names are susceptible to this ambiguity and run the risk of providing imprecise or incomplete sets of data to subsequent downstream processes.

Sets of related scientific names may be used, as in today’s array of taxonomic name servers, to improve recall in search and retrieval for information tied to a taxon. The ambiguity of scientific names that occurs when the same name refers to two distinct, or overlapping taxa, however means that, in many cases, a single name returns an imprecise result and this is something that cannot be rectified through the use of name services.

Comprehensive taxonomic thesauri are required to model the relationships between names and taxa. Nomenclatural databases that currently capture the objective syntactic properties of names could improve their relevance by cataloging nomenclatural synonyms, as attempted in Index Animalium. Effectively modeling semantics requires a clean division between these syntactic aspects of taxonomy and the subsequent subjective processes that result in changes in circumscription (Lepage et al. 2014). An ideal system enables the identification, modeling and exposure of a complete array of circumscription changes for any and all taxa and are coupled with services that allow these to be embedded within informatics processes (Chem et al. 2014, Tuominen et al. 2011).
